# Association of Central Obesity With All Cause and Cause-Specific Mortality in US Adults: A Prospective Cohort Study

**DOI:** 10.3389/fcvm.2022.816144

**Published:** 2022-01-28

**Authors:** Pengcheng Huai, Jian Liu, Xing Ye, Wen-Qing Li

**Affiliations:** ^1^Shandong Provincial Hospital for Skin Diseases & Shandong Provincial Institute of Dermatology and Venereology, Shandong First Medical University & Shandong Academy of Medical Sciences, Jinan, China; ^2^Key Laboratory of Carcinogenesis and Translational Research (Ministry of Education/Beijing), Department of Cancer Epidemiology, Peking University Cancer Hospital & Institute, Beijing, China

**Keywords:** central obesity, mortality, cardiovascular diseases, cohort study, NHANES

## Abstract

**Background:**

Previous data on the association between central obesity and mortality are controversial. The aim of this study was to determine the associations between central obesity, as measured by the waist-to-height ratio (WtHR) and waist circumference (WC), with all cause and cause-specific mortality in U.S. adults.

**Methods:**

The study subjects comprised a nationally representative sample of 33,569 adults >20 years of age who were recruited in the National Health and Nutrition Examination Survey between 1999 and 2014. Anthropometric data, including weight, height, and WC, were measured at each of the eight waves using consistent methodology. Death and underlying causes of death were ascertained through 31 December 2015. The association between central obesity and mortality were determined using weighted Cox proportional hazards regression models.

**Results:**

A total of 4013 deaths occurred during a median follow-up of 7.33 years (263,029 person-years). Compared with the subjects in WtHR tertile 1, the subjects in tertiles 2 and 3 were at a higher risk of mortality from all-cause (tertile 2-hazard ratio [HR]: 1.29; 95% confidence interval [CI]: 1.13–1.47; tertile 3-HR: 1.96; 95% CI: 1.64–2.34) and cardiovascular diseases [CVDs] (tertile 2-HR: 1.40; 95% CI: 1.09–1.79; tertile 3-HR: 2.00; 95% CI: 1.47–2.73). Similarly, compared with the subjects in WC tertile 1, the subjects in tertiles 2 and 3 were at a higher risk of mortality from all-cause (tertile 2-HR: 1.15; 95% CI: 1.00–1.31; tertile 3-HR: 1.39; 95% CI: 1.15–1.67) and CVD (tertile 2-HR: 1.48; 95% CI: 1.14–1.93; tertile 3-HR: 1.74; 95% CI: 1.26–2.42). Restricted cubic spline analyses revealed an S-shaped and linear dose-relationship between WtHR and WC with all-cause mortality. Moreover, a WtHR> 0.58 or a WC > 0.98m was shown to be a risk factor for all-cause mortality.

**Conclusions:**

Central obesity was significantly associated with increased risk of all-cause and CVD-related mortality, especially heart diseases-related mortality, even among normal weight adults. In addition to weight control, guideline designer should provide recommendations for people to decrease abdominal fat accumulation, in their effort to reduce mortality risk in later life.

## Introduction

Central obesity has become a major public health problem in the United States (U.S.), and the estimated prevalence of central obesity in U.S. adults increased from 43.5% in men and 64.7% in women between 2011 and 2012, to 50.1 and 72.5%, respectively, in 2020 (1, 2). Indeed, collective evidence indicated that central obesity, as usually reflected by the waist circumference (WC), waist-to-hip ratio (WHR), and waist-to-height ratio (WtHR), is significantly associated with a higher risk of chronic diseases, such as cardiovascular disease (CVD) or cancer, and the associations are independent of the Body Mass Index (BMI) (3–5); however, the evidence pertaining to the association between central obesity and mortality is conflicting. The majority of studies have shown a positive J-shaped relationship (6–8), but few studies have reported a negative association (9, 10). Furthermore, most studies evaluating the association between central obesity and mortality in select populations such as postmenopausal women, those with chronic health conditions, older adults, or participants located in a given city, rather than a nationally-representative general population (11–14). Additionally, some large scale cohort studies have used self-reported measures rather than technician-measured data to perform analyses, which could result in inaccurate assessment of central obesity because participants tend to over-report their height (6, 15, 16). In addition, several cohort studies did not remove subjects with serious illnesses at baseline to limit the effect of reverse causality (9). Recently, a dose-response meta-analysis involving 72 prospective cohort studies reported a nearly J-shaped association between central obesity indices and all cause mortality (17); however, the potential effects of reverse causality were not restricted. Furthermore, the underlying confounding factors, such as cigarette smoking, were not adjusted in this analysis due an inability to obtain raw data from each of the included studies.

Because the existing evidence is insufficient and controversial regarding to the association between central obesity and mortality, we conducted this study using a nationally representative sample of U.S. adults with precisely measured data to assess the association between central obesity and all-cause and cause-specific mortality.

## Methods

### Study Design and Population

We used data from the National Health and Nutrition Examination Survey (NHANES), an ongoing national 2-year-cycle cross-sectional survey conducted by the US Centers for Disease Control and Prevention. Potential participants were selected by a complex, stratified, multistage probability sampling design and were representative of the civilian, non-institutionalized resident population of the U.S. Participants were first interviewed in their homes to collect demographics data and basic health information, then the participants underwent a standardized physical examination in a specially equipped mobile examination center (MEC) for the collection of other health data, such as anthropometric, and laboratory measurements. Written informed consent was provided from all participants or proxies, and the protocol was approved by the Ethics Review Board at the National Center for Health Statistics (NCHS). Detailed information on the design, data collection procedures and weighting are described elsewhere (18). This study included data from representative adults aged >20 years of age who participated in the 8 cycles (1999–2000 to 2013–2014) of the NHANES, with linkage to the National Death Index to 31 December 2015. Among the 43,793 subjects, 10,224 were excluded because of pregnancy (*n* = 1,416), missing data on height, weight, or WC (*n* = 4,543), missing data on mortality status (*n* = 50), and missing data on covariates (demographic information, lifestyle variables, or chronic health conditions, *n* = 4,215), resulting in a final analytical sample of 33,569 adults.

### Anthropometric Measures

Baseline anthropometric information, including weight, height, and WC, were measured during mobile physical examinations in the MEC by trained staff (19). Weight was measured to the nearest 0.1 kg using a digital weight scale with the participants wearing a standard MEC examination gown, consisting of a disposable shirt, pants, and slippers. Height was measured to the nearest 0.1 cm using a stadiometer with a fixed vertical backboard and an adjustable head piece. Participants were instructed to stand with the back of the head, shoulder blades, buttocks, and heels in contact with the vertical backboard. WC was measured to the nearest 0.1 cm using a tape at the uppermost lateral border of the ilium. The WtHR was calculated by dividing the WC by height (both in centimeters).

### Mortality

The mortality status of the participants was ascertained by probabilistic matching the NHANES database to the National Death Index records through 31 December, 2015 based on a unique sequence number. Detailed information on linkage methods is available from the NCHS (20).The accuracy of information in the National Death Index records was validated using mortality-linked data from the NHANES I epidemiologic follow-up survey; 98.5% of participants were classified correctly (21). The 10th revision of the International Classification of Diseases (ICD-10) was used to ascertain the underlying cause of death. We identified deaths from all causes, CVDs (I00–I09, I11, I13, I20–I51, I60–I69), cancers (C00–C97), chronic lower respiratory diseases (J40–J47), diabetes (E10–E14), and other causes. The follow-up duration was defined as the interval from the examination date in the NHANES MEC to the date of death for decedents or to 31 December 2015 for those who were censored.

### Covariates Assessment

Covariates of this study involved major demographic characteristics, lifestyle factors, and personal history of chronic diseases, which may be associated with all-cause mortality based on previous literature (22). Demographic and lifestyle variables including age, gender, race/ethnicity, education, marital status, and smoking status, were collected during household interviews, while alcohol intake was obtained during the mobile physical examination in the MEC. Race/ethnicity was classified as Hispanic, non-Hispanic white, non-Hispanic black, or other race. Educational level was categorized as grades 0–12, high school/general equivalency diploma (GED), and some college or above. Marital status was categorized as married, widowed/divorced/separated, never married, and living with a partner. BMI was defined as the weight in kg divided by the height in meters squared, and categorized into normal weight (18.5– <25 kg/m^2^), overweight (25– <30 kg/m^2^), and obesity (≥30 kg/m^2^). Participants were categorized as never, former, and current smokers based on their response to questions about smoking at least 100 cigarettes in their lifetime and whether they were currently smoking. The amount of alcohol intake was classified based on three queries that questioned whether the participant had at least 12 alcohol drinks in any given year (a drink means a 12 ounce beer, a 4 ounce glass of wine, or an ounce of liquor), the amount of days of drinking in the past year and the number of drinks per day on a given drinking day (23). Participants were categorized into the following four alcohol consumption groups: lifetime abstainers (<12 drinks in any given year), former drinkers (≥12 drinks in a previous year), current light to moderate drinkers (current use of <14 drinks/week for men or <7 drinks/week for women), and current heavy drinkers (≥14 drinks/week for men or ≥7 drinks/week for women). Information on personal history of major physician-diagnosed chronic health conditions, including hypertension, diabetes, heart disease (congestive heart failure, coronary heart disease, angina/angina pectoris, or heart attack), stroke, and cancer was collected based on the self-report of participants.

### Statistical Analysis

All statistical analyses for the complex sampling design of NHANES by using sample weights, strata, and primary sampling units as specified in the guidelines for analyzing NHANES data (24). The baseline characteristics of participants across the WtHR and WC tertiles were compared using a χ^2^ test. We calculated person-years from baseline to the date of death, or 31 December 2015, whichever came first. We tested the proportional hazards assumption by creating interaction terms of exposures and follow-up time and did not identify any violations. We used Cox proportional hazards models to calculated hazard ratios (HRs) and 95% confidence intervals (CIs) for the risk of all cause and cause-specific mortality associated with different measures of central obesity. Three multivariable models were constructed: Model 1 was adjusted for age, gender, race/ethnicity, education, marital status, and BMI; Model 2 was additionally adjusted for smoking status, and alcohol intake; and Model 3 was additionally adjusted for chronic health conditions, including hypertension, diabetes, heart disease, stroke, and cancer. Restricted cubic splines (RCSs) with five knots at 5, 25, 50, 75, and 95th percentiles were used to determine the dose-response relationships between the WtHR and WC, and with all-cause mortality after adjusting for all the covariates. We used a stratified analysis to determine whether the association between central obesity and mortality was modified by major baseline variables, including age, gender, race/ethnicity, education, marital status, BMI, smoking status, alcohol intake, and chronic health conditions. Based on sensitivity analysis, we excluded participants who had follow-up evaluations of <2 years duration and removed participants with any chronic health conditions at baseline to minimize potential reverse causation. All analyses were performed using SAS 9.3 (SAS Institute, Inc., Cary, NC, USA). A two tailed *P*-value < 0.05 was considered to be statistically significant.

## Results

Table 1 shows the baseline characteristics, including demographic factors, lifestyles and personal history of major chronic diseases, of 33,569 eligible adults according to the WtHR and WC tertiles. There were statistically significant differences in each baseline characteristic across the three categories of the WtHR and WC. Participants in WtHR and WC tertile 3 were more likely to be older, non-Hispanic blacks, widowed/divorced/separated, obese, former smokers, lifetime abstainers or former drinkers, have a high school/GED education, and have more chronic conditions when compared with the subjects in tertile 1.

**Table 1:** Characteristics of study adults according to baseline measures of the WtHR and WC in the National Health and Nutrition Examination Survey, 1999–2014.

**Characteristics**	**WtHR**			***P*-values**	**WC**			***P*-values**
	**Tertile 1**	**Tertile 2**	**Tertile 3**		**Tertile 1**	**Tertile 2**	**Tertile 3**	
**Age group (years)**
20–39	5,521(51.66)	2,998(29.70)	2,435(24.92)	<0.001	5,331(51.32)	2,996(30.80)	2,627(26.13)	<0.001
40–59	3,435(34.92)	3,925(42.91)	3,762(41.09)		3,295(33.75)	3,879(41.87)	3,948(42.98)	
≥60	2,233(13.42)	4,265(27.39)	4,993(33.98)		2,549(14.93)	4,326(27.33)	4,618(30.89)	
**Sex**
Men	6,048(50.74)	6,314(55.54)	4,633(42.12)	<0.001	5,664(46.60)	5,678(50.69)	5,653(52.15)	<0.001
Women	5,141(49.26)	4,876(44.46)	6,557(57.88)		5,511(53.40)	5,523(49.31)	5,540(47.85)	
**Race/ethnicity**
Hispanic	2,092(10.23)	3,208(14.93)	3,132(14.03)	<0.001	2,650(12.93)	3,230(14.62)	2,552(11.14)	<0.001
Non-Hispanic White	5,624(72.32)	5,233(70.33)	5,216(69.95)		5,143(68.85)	5,267(70.85)	5,663(73.39)	
Non-Hispanic Black	2,384(10.43)	2,036(9.32)	2,432(12.23)		2,191(10.19)	2,050(9.49)	2,611(12.18)	
Other race	1,089(7.02)	713(5.42)	410(3.79)		1,191(8.03)	654(5.04)	367(3.29)	
**Education**
Grades 0–12	2,246(13.34)	3,243(18.75)	3,640(21.70)	<0.001	2,684(15.69)	3,300(18.81)	3,145(18.46)	<0.001
High school/GED	2,458(21.37)	2,623(24.48)	2,788(27.27)		2,435(21.73)	2,582(23.77)	2,852(27.15)	
Some college or above	6,485(65.29)	5,324(56.77)	4,762(51.03)		6,056(62.58)	5,319(57.42)	5,196(54.39)	
**Marital status**
Married	5,503(52.70)	6,623(62.94)	5,974(57.67)	<0.001	5,464(51.81)	6,445(61.44)	6,191(59.78)	<0.001
Widowed/Divorced/Separated	1,918(14.43)	2,475(18.67)	3,086(23.11)		2,057(15.36)	2,626(19.29)	2,796(20.83)	
Never married	2,819(24.32)	1,361(12.10)	1,523(13.53)		2,731(24.38)	1,401(12.80)	1,571(13.55)	
Living with partner	949(8.55)	731(6.29)	607(5.69)		923(8.45)	729(6.47)	635(5.84)	
**Body mass index category**
Normal	8,319(73.49)	1,507(10.80)	50(0.31)	<0.001	8,415(76.90)	1,446(12.58)	15(0.14)	<0.001
Overweight	2,789(25.84)	7,274(65.14)	1,686(12.21)		2,692(22.61)	7,378(67.03)	1,679(14.93)	
Obesity	81(0.67)	2,409(24.06)	9,454(87.48)		68(0.49)	2,377(20.39)	9,499(84.93)	
**Smoking status**
Never	6,044(54.22)	5,781(51.36)	5,903(51.73)	<0.001	6,194(54.65)	5,899(52.82)	5,635(49.97)	<0.001
Former	2,150(19.66)	3,176(27.99)	3,270(28.92)		2,132(19.63)	3,039(25.95)	3,425(30.32)	
Current	2,995(26.12)	2,233(20.65)	2,017(19.35)		2,849(25.72)	2,263(21.23)	2,133(19.71)	
**Alcohol intake**
Lifetime abstainers	2,630(19.42)	3,048(23.36)	3,932(31.19)	<0.001	2,913(21.29)	3,237(24.50)	3,460(27.14)	<0.001
Former drinker	826(6.06)	1,324(9.54)	1,674(13.41)		886(6.17)	1,283(9.22)	1,655(13.07)	
Light to moderate	6,522(62.52)	5,776(56.20)	4,876(48.22)		6,228(60.73)	5,687(55.77)	5,259(51.65)	
Heavy	1211(12.00)	1042(10.90)	708(7.18)		1148(11.81)	994(10.51)	819(8.14)	
**Chronic conditions**
Hypertension	2,034(14.99)	3,909(31.60)	5,776(48.56)	<0.001	2,124(15.36)	3,935(30.42)	5,660(46.87)	<0.001
Diabetes	411(2.30)	1,084(6.56)	2,339(17.21)	<0.001	473(2.67)	1,135(6.37)	2,226(15.88)	<0.001
Heart disease	472(3.01)	960(6.93)	1,375(10.61)	<0.001	520(3.30)	941(6.70)	1,346(9.97)	<0.001
Stroke	190(1.23)	379(2.50)	565(4.00)	<0.001	209(1.33)	417(2.75)	508(3.42)	<0.001
Cancer	760(6.89)	1,133(10.00)	1,243(11.52)	<0.001	774(7.03)	1,131(9.88)	1,231(11.13)	<0.001

*Values are n (percentages) unless stated otherwise*.

*WtHR, waist-to-height ratio; WC, waist circumference; GED, general equivalency diploma*.

*WtHR: Tertile 1, ≤ 0.54; Tertile 2, 0.55-0.62; Tertile 3, ≥0.63*.

*WC: Tertile 1, Men ≤ 0.93 cm, Women ≤ 0.88cm; Tertile 2, Men 0.94-1.05 cm, Women 0.89–1.02 cm; Tertile 3, Men ≥1.06cm, Women ≥1.03 cm*.

During 263,029 person-years of follow-up (median follow-up, 7.33 years; maximum follow-up, 16.75 years), 4013 deaths occurred, including 863 deaths from CVD, 935 from cancer, 150 from chronic lower respiratory tract diseases, 103 from diabetes, and 1962 from other causes.

The associations between WtHR and WC with all-cause and cause-specific mortality are shown in Table 2. Participants in WtHR or WC tertiles 2 or 3 were at higher risk for all cause and CVD mortality compared with the subjects in tertile 1 across the 3 models. In the fully adjusted model, the multivariable-adjusted HRs for all-cause and CVD-related mortality for participants in WtHR tertile 3 were 1.96 (95% CI, 1.64–2.34) and 2.00 (95% CI, 1.47–2.73), respectively, while the HRs for the subjects in WtHR tertile 2 were 1.29 (95% CI, 1.13–1.47) and 1.40 (95% CI, 1.09–1.79), respectively, compared with the subjects in WtHR tertile 1. After adjustment for all confounders, the HRs for all-cause and CVD mortality for the subjects in WC tertile 3 were 1.39 (95% CI, 1.15–1.67) and 1.74 (95% CI, 1.26–2.42), respectively, while the HRs for the subjects in WC tertile 2 were 1.15 (95% CI, 1.00–1.31) and 1.48 (95% CI, 1.14–1.93), respectively, compared with WC tertile 1. In Model 3, participants in WtHR tertile 3 had a 71% higher risk of death from cancer (HR, 1.71, 95% CI, 1.18–2.47) and a 2.52-fold higher risk of death from chronic lower respiratory tract diseases (HR, 2.52, 95% CI, 1.13–5.63), compared with the subjects in WtHR tertile 1. The individuals in WC tertile 3 had a 3.62-fold higher risk of death from chronic lower respiratory tract diseases (HR, 3.62, 95% CI, 1.72–7.59), compared with those in WC tertile 1. A similar association existed between the WtHR and other causes of mortality across the three models, as well as WC and other causes of mortality in Model 1. The increasing trend in HRs with the increase in the WtHR or WC was demonstrated by the above models; however, the associations between the WtHR and WC with diabetes were not consistently significant across the three models.

**Table 2:** Associations between the WtHR and WC with all-cause and cause-specific mortality.

**Cause of death**	**WtHR**				**WC**			
	**Tertile 1**	**Tertile 2**	**Tertile 3**	**P for trend**	**Tertile 1**	**Tertile 2**	**Tertile 3**	**P for trend**
**All causes**								
No. of deaths	935	1,433	1,645		1,035	1,476	1,502	
Model 1	1.00	1.37(1.21–1.55)	2.37(2.01–2.79)	<0.001	1.00	1.19(1.06–1.35)	1.63(1.37–1.94)	<0.001
Model 2	1.00	1.37(1.21–1.55)	2.32(1.97–2.73)	<0.001	1.00	1.19(1.05–1.34)	1.57(1.32–1.88)	<0.001
Model 3	1.00	1.29(1.13–1.47)	1.96(1.64–2.34)	<0.001	1.00	1.15(1.00–1.31)	1.39(1.15–1.67)	<0.001
**Cardiovascular diseases**								
No. of deaths	169	325	369		188	341	334	
Model 1	1.00	1.55(1.22–1.96)	2.70(2.03–3.57)	<0.001	1.00	1.52(1.19–1.95)	2.10(1.52–2.91)	<0.001
Model 2	1.00	1.53(1.21–1.94)	2.59(1.95–3.45)	<0.001	1.00	1.52(1.18–1.94)	2.06(1.49–2.85)	<0.001
Model 3	1.00	1.40(1.09–1.79)	2.00(1.47–2.73)	<0.001	1.00	1.48(1.14–1.93)	1.74(1.26–2.42)	0.001
**Cancer**								
No. of deaths	232	330	373		241	345	349	
Model 1	1.00	1.13(0.86–1.49)	1.78(1.24–2.56)	0.002	1.00	1.03(0.80–1.32)	1.15(0.80–1.67)	0.432
Model 2	1.00	1.15(0.88–1.51)	1.79(1.24–2.58)	0.002	1.00	1.02(0.79–1.30)	1.09(0.74–1.59)	0.656
Model 3	1.00	1.12(0.85–1.47)	1.71(1.18–2.47)	0.003	1.00	0.98(0.76–1.27)	1.01(0.68–1.50)	0.947
**Chronic lower respiratory tract diseases**								
No. of deaths	42	53	55		42	56	52	
Model 1	1.00	0.98(0.59–1.64)	2.42(1.15–5.11)	0.041	1.00	1.30(0.76–2.20)	4.26(2.10–8.62)	<0.001
Model 2	1.00	1.09(0.64–1.84)	2.73(1.27–5.86)	0.022	1.00	1.28(0.77–2.15)	3.89(1.90–7.94)	<0.001
Model 3	1.00	1.05(0.62–1.78)	2.52(1.13–5.63)	0.043	1.00	1.26(0.75–2.12)	3.62(1.72–7.59)	0.002
**Diabetes mellitus**								
No. of deaths	11	32	60		17	39	47	
Model 1	1.00	2.68(1.00–7.13)	6.04(2.15–16.96)	<0.001	1.00	1.76(0.72–4.31)	2.27(0.98–5.24)	0.051
Model 2	1.00	2.56(0.97–6.76)	5.41(0.94–15.13)	0.001	1.00	1.73(0.71–4.24)	2.18(0.93–5.14)	0.069
Model 3	1.00	1.44(0.53–3.96)	1.59(0.50–5.10)	0.505	1.00	1.33(0.51–3.47)	0.97(0.37–2.57)	0.673
**Other causes**								
No. of deaths	481	693	788		547	695	720	
Model 1	1.00	1.45(1.20–1.74)	2.48(1.92–3.22)	<0.001	1.00	1.14(0.95–1.36)	1.57(1.24–2.00)	<0.001
Model 2	1.00	1.44(1.19–1.73)	2.40(1.85–3.13)	<0.001	1.00	1.13(0.95–1.36)	1.55(1.22–1.97)	<0.001
Model 3	1.00	1.36(1.12–1.65)	2.05(1.58–2.67)	<0.001	1.00	1.10(0.91–1.32)	1.37(1.08–1.74)	0.009

*Values are n or weighted hazard ratio (95% confidence interval) unless stated otherwise*.

*Model 1: Adjusted for sex, age, race/ethnicity, education, marital status and BMI*.

*Model 2: Model 1+smoking status, and alcohol intake*.

*Model 3: Model 2+chronic conditions (including hypertension, diabetes, heart disease, stroke, and cancer)*.

*WtHR, waist-to-height ratio; WC, waist circumference*.

*WtHR: Tertile 1, ≤ 0.54; Tertile 2, 0.55-0.62; Tertile 3, ≥0.63*.

*WC: Tertile 1, Men ≤ 0.93 cm, Women ≤ 0.88cm; Tertile 2, Men 0.94–1.05 cm, Women 0.89–1.02 cm; Tertile 3, Men ≥1.06cm, Women ≥1.03 cm*.

We further divided CVDs into heart diseases (I00–I09, I11, I13, I20–I51) and cerebrovascular diseases (I60–I69). The association between WtHR and heart disease-related mortality was significant across the 3 models, and the effect magnitude increased with elevated WtHR (Table 3). In the fully-adjusted model, the HRs for heart diseases-related mortality for participants in tertile 2 or 3 were 1.41 (95% CI, 1.06–1.87) and 2.16 (95% CI, 1.51–3.11), respectively, compared with the subjects in WtHR tertile 1. The relationships between WC and heart, and cerebrovascular disease-related mortality were significant across the 3 models, and the HRs increased in parallel with the increase in WC (Table 3). After adjustment for all variables, the effect sizes for heart and cerebrovascular diseases-related mortality for the subjects in WC tertile 3 of were 1.39 (95% CI, 1.15–1.67) and 1.74 (95% CI, 1.26–2.42), respectively, and the HRs for the subjects in WC tertile 2 were 1.15 (95% CI, 1.00–1.31) and 1.48 (95% CI, 1.14–1.93), respectively, compared with the subjects in WC tertile 1.

**Table 3:** Associations between theWtHR and WC with heart and cerebrovascular diseases-specific mortality.

**Cause of death**	**WtHR**				**WC**			
	**Tertile 1**	**Tertile 2**	**Tertile 3**	***P* for trend**	**Tertile 1**	**Tertile 2**	**Tertile 3**	***P* for trend**
**Heart diseases**								
No. of deaths	135	265	305		156	271	278	
Model 1	1.00	1.56(1.19–2.04)	2.95(2.11–4.12)	<0.001	1.00	1.19(1.06–1.35)	1.63(1.37–1.94)	<0.001
Model 2	1.00	1.56(1.19–2.04)	2.85(2.04–4.00)	<0.001	1.00	1.19(1.05–1.34)	1.57(1.32–1.88)	<0.001
Model 3	1.00	1.41(1.06–1.87)	2.16(1.51–3.11)	<0.001	1.00	1.15(1.00–1.31)	1.39(1.15–1.67)	0.007
**Cerebrovascular diseases**								
No. of deaths	34	60	64		32	70	56	
Model 1	1.00	1.49(0.98–2.28)	1.81(0.94–3.47)	0.080	1.00	1.52(1.19–1.95)	2.10(1.52–2.91)	0.029
Model 2	1.00	1.45(0.95–2.21)	1.69(0.88–3.25)	0.117	1.00	1.52(1.18–1.94)	2.06(1.49–2.85)	0.026
Model 3	1.00	1.36(0.88–2.09)	1.41(0.72–2.78)	0.318	1.00	1.48(1.14–1.93)	1.74(1.26–2.42)	0.053

*Values are n or weighted hazard ratio (95% confidence interval) unless stated otherwise*.

*Model 1: Adjusted for sex, age, race/ethnicity, education, marital status and BMI*.

*Model 2: Model 1+smoking status, and alcohol intake*.

*Model 3: Model 2+chronic conditions (including hypertension, diabetes, heart disease, stroke, and cancer)*.

*WtHR, waist-to-height ratio; WC, waist circumference*.

*WtHR: Tertile 1, ≤ 0.54; Tertile 2, 0.55–0.62; Tertile 3, ≥0.63*.

*WC: Tertile 1, Men ≤ 0.93 cm, Women ≤ 0.88cm; Tertile 2, Men 0.94-1.05 cm, Women 0.89-1.02 cm; Tertile 3, Men ≥1.06cm, Women ≥1.03 cm*.

*Heart diseases:I00–I09, I11, I13, I20–I51*.

*Cerebrovascular diseases: I60–I69*.

**Figure 1:**
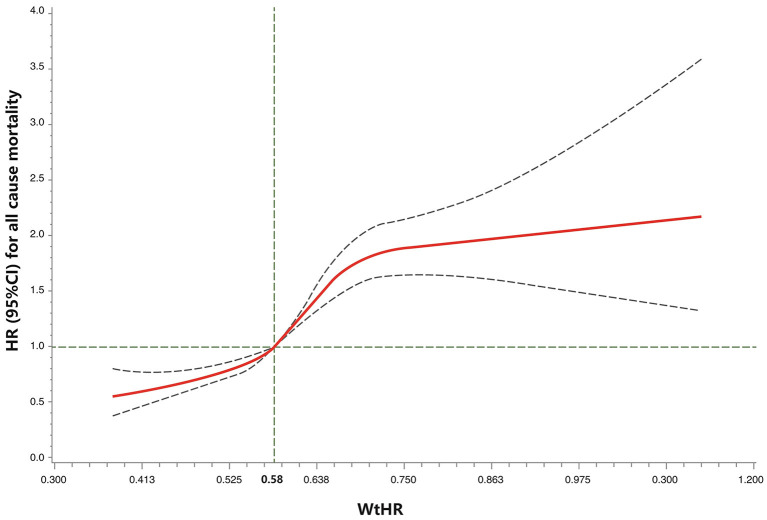
Restricted cubic spline model of the association between the WtHR and all-cause mortality. Legend: Adjusted for demographics, lifestyle factors, body mass index, and chronic health conditions. The solid curve represents the HRs, and the dashed curves represent the 95% CIs. *P*_overall_ < 0.01, *P*_nonlinear_ < 0.01. WtHR, waist-to-height ratio.

**Figure 2:**
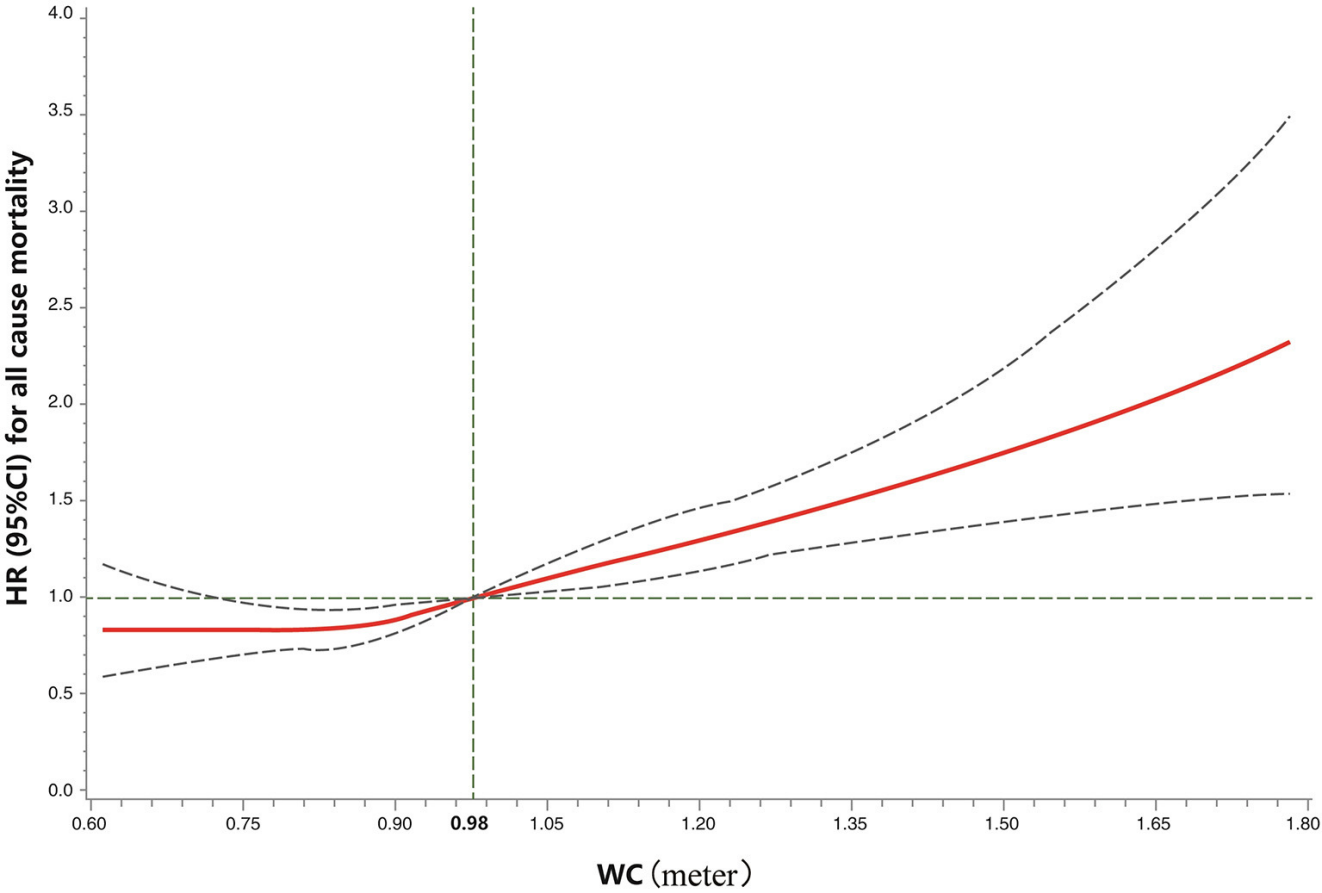
Restricted cubic spline model of the association between the WC and all-cause mortality. Legend: Adjusted for demographics, lifestyle factors, body mass index, and chronic health conditions. The solid curve represents the HRs, and the dashed curves represent the 95% CIs. *P*_overall_ < 0.01, *P*_nonlinear_ = 0.52. WC, waist circumference (meter).

The RCS analyses showed that the WtHR was associated with all-cause mortality in an S-shaped non-linear manner in the fully-adjusted model (*P*_overall_ < 0.01, *P*_nonlinear_ < 0.01; Figure 1). The risk of all-cause mortality increased with the increase in the WtHR. Specifically, a WtHR>0.58 was shown to be a risk factor of all-cause mortality. The HRs of all-cause mortality increased with the increase in WC in a linear dose-responsive manner (*P*_overall_ < 0.01, P_nonlinear_ = 0.52; Figure 2). Specifically, a WC > 0.98 m was shown to be a risk factor for all-cause mortality. These results were consistent with the results obtained when the WtHR and WC were treated as category variables in Table 2.

Based on the stratified analyses (Table 4), the association between WtHR tertile 3 and all-cause mortality was stronger among middle-aged adults (vs. younger or older adults), adults with a high school education (vs. adults with a grade 0–12 education or some college or above education), healthy adults (vs. adults with hypertension or stroke or cancer).The effect sizes between WC tertile 3 and all-cause mortality were stronger among middle-aged adults (vs. younger or older adults), adults with a higher education (vs. lower education), healthy adults (vs. adults with hypertension or cancer). The increasing trend in HRs with the increase in the WtHR or WC was present in most of the subgroups. In addition, the forest plots for the WtHR or WC and all-cause and cause-specific mortality in the normal weight and overweight subgroups, as measured by BMI, are shown in Supplementary Figures 1, 2. The forest plots for the obesity subgroup are not shown because the number of deaths in WtHR or WC tertile 1 in obese subjects was small, which resulted in infinite HRs. In the normal weight subgroup, we found positive associations between WtHR tertile 2 or 3 with all-cause mortality, and CVD, diabetes, and other causes of mortality. The associations between WC tertile 2 or 3 with all-cause mortality, and CVD, and chronic lower respiratory tract diseases-related mortality were positive. In the overweight subgroup, positive associations were shown between WtHR tertile 2 or 3 with all-cause mortality, and CVD, cancer, diabetes, and other causes of mortality. The association between WC tertile 3 and chronic lower respiratory tract disease-related mortality was positive.

**Table 4:** Stratified analysis of associations between the WtHR and WC with all-cause mortality.

**Subgroups**	**WtHR**					**WC**				
	**Tertile 1**	**Tertile 2**	**Tertile 3**	***P* for trend**	***P* for interaction**	**Tertile 1**	**Tertile 2**	**Tertile 3**	***P* for trend**	***P* for interaction**
**Age group (years)**										
20–39	1.00	1.09 (0.59–2.01)	1.25 (0.56–2.82)	0.580	<0.001	1.00	1.06 (0.53–2.12)	1.58 (0.75–3.33)	0.190	<0.001
40–59	1.00	1.31 (1.01–1.70)	2.48 (1.66–3.68)	<0.001		1.00	1.17 (0.87–1.57)	1.77 (1.19–2.64)	0.004	
≥60	1.00	1.19 (1.03–1.37)	1.74 (1.43–2.13)	<0.001		1.00	1.09 (0.96–1.25)	1.22 (1.02–1.47)	0.031	
**Sex**										
Men	1.00	1.40 (1.18–1.66)	2.37 (1.82–3.08)	<0.001	0.312	1.00	1.22 (1.01–1.47)	1.57 (1.23–2.01)	<0.001	0.507
Women	1.00	1.22 (1.03–1.45)	1.62 (1.29–2.04)	<0.001		1.00	1.09 (0.92–1.29)	1.25 (0.98–1.59)	0.070	
**Race/ethnicity**										
Hispanic	1.00	1.04 (0.77–1.40)	1.59 (1.04–2.44)	0.021	0.491	1.00	0.96 (0.75–1.21)	1.22 (0.86–1.72)	0.230	0.345
Non-Hispanic White	1.00	1.37 (1.17–1.60)	2.16 (1.74–2.68)	<0.001		1.00	1.18 (1.02–1.37)	1.42 (1.15–1.75)	0.001	
Non-Hispanic Black	1.00	1.25 (0.93–1.68)	2.01 (1.35–3.00)	<0.001		1.00	1.23 (0.95–1.60)	1.43 (1.04–1.97)	0.030	
Other race	1.00	0.88 (0.46–1.69)	0.71 (0.26–1.97)	0.525		1.00	0.85 (0.46–1.56)	1.06 (0.36–3.15)	0.951	
**Education**										
Grades 0–12	1.00	0.97 (0.81–1.16)	1.48 (1.13–1.94)	0.003	<0.001	1.00	1.00 (0.83–1.20)	1.16 (0.92–1.45)	0.199	0.002
High school/GED	1.00	1.45 (1.11–1.89)	2.52 (1.67–3.79)	<0.001		1.00	1.11 (0.83–1.47)	1.41 (0.97–2.05)	0.056	
Some college or above	1.00	1.47 (1.21–1.80)	2.13 (1.63–2.77)	<0.001		1.00	1.32 (1.07–1.64)	1.70 (1.26–2.29)	<0.001	
**Marital status**										
Married	1.00	1.32 (1.09–1.59)	2.02 (1.57–2.60)	<0.001	0.635	1.00	1.21 (1.00–1.46)	1.51 (1.17–1.95)	0.001	0.349
Widowed/Divorced/Separated	1.00	1.15 (0.96–1.38)	1.77 (1.42–2.19)	<0.001		1.00	1.05 (0.88–1.25)	1.31 (1.01–1.70)	0.044	
Never married	1.00	1.39 (0.84–2.29)	1.96 (0.91–4.21)	0.086		1.00	1.12 (0.66–1.88)	1.08 (0.56–2.09)	0.804	
Living with partner	1.00	1.69 (0.90–3.17)	2.07 (0.81–5.34)	0.114		1.00	1.70 (0.93–3.10)	1.48 (0.57–3.88)	0.389	
**Body mass index category**										
Normal weight	1.00	1.18 (1.00–1.39)	1.66 (1.04–2.64)	0.021	0.308	1.00	1.20 (1.04–1.40)	0.86 (0.48–1.52)	0.034	0.197
Overweight	1.00	1.36 (1.07–1.74)	2.01 (1.53–2.63)	<0.001		1.00	1.09 (0.88–1.35)	1.24 (0.97–1.58)	0.046	
Obesity	1.00	0.85 (0.11–6.45)	1.46 (0.20–10.81)	<0.001		1.00	0.49 (0.17–1.48)	0.68 (0.22–2.15)	0.023	
**Smoking status**										
Never	1.00	1.44 (1.19–1.73)	1.92 (1.54–2.39)	<0.001	0.849	1.00	1.13 (0.94–1.35)	1.28 (1.01–1.61)	0.036	0.197
Former	1.00	1.26 (1.04–1.53)	1.96 (1.49–2.59)	<0.001		1.00	1.30 (1.03–1.64)	1.60 (1.18–2.17)	0.003	
Current	1.00	1.02 (0.75–1.39)	1.79 (1.09–2.95)	0.023		1.00	0.93 (0.68–2.26)	1.31 (0.80–2.14)	0.306	
**Alcohol intake**										
Lifetime abstainers	1.00	1.19 (0.97–1.47)	1.68 (1.26–2.23)	<0.001	0.263	1.00	1.05 (0.85–1.28)	1.26 (0.95–1.66)	0.098	0.484
Former drinker	1.00	1.41 (1.08–1.85)	2.06 (1.46–2.92)	<0.001		1.00	1.30 (0.99–1.70)	1.76 (1.18–2.60)	0.006	
Light to moderate	1.00	1.26 (1.04–1.52)	1.97 (1.43–2.70)	<0.001		1.00	1.18 (0.96–1.45)	1.45 (1.08–1.96)	0.014	
Heavy	1.00	1.27 (0.86–1.87)	2.14 (1.16–3.94)	0.016		1.00	0.94 (0.64–1.38)	0.90 (0.50–1.60)	0.705	
**Hypertension**										
Yes	1.00	1.18 (0.99–1.40)	1.68 (1.32–2.15)	<0.001	<0.001	1.00	1.08 (0.89–1.31)	1.26 (0.99–1.60)	0.046	0.001
No	1.00	1.30 (1.09–1.57)	2.20 (1.69–2.87)	<0.001		1.00	1.15 (0.99–1.34)	1.53 (1.21–1.93)	<0.001	
**Diabetes**										
Yes	1.00	0.77 (0.53–1.13)	1.02 (0.65–1.59)	0.571	0.120	1.00	1.12 (0.81–1.54)	1.22 (0.82–1.81)	0.318	0.838
No	1.00	1.39 (1.23–1.57)	2.20 (1.83–2.63)	<0.001		1.00	1.15 (1.01–1.31)	1.41 (1.14–1.74)	0.002	
**Heart disease**										
Yes	1.00	1.45 (1.17–1.80)	1.86 (1.39–2.50)	<0.001	0.245	1.00	1.48 (1.14–1.93)	1.51 (1.08–2.12)	0.032	0.434
No	1.00	1.24 (1.08–1.43)	2.00 (1.65–2.42)	<0.001		1.00	1.05 (0.92–1.20)	1.35 (1.09–1.68)	0.006	
**Stroke**										
Yes	1.00	1.29 (0.89–1.86)	1.35 (0.87–2.10)	0.209	0.033	1.00	1.23 (0.84–1.81)	1.22 (0.75–2.01)	0.473	0.195
No	1.00	1.29 (1.11–1.48)	2.05 (1.68–2.51)	<0.001		1.00	1.13 (0.98–1.30)	1.41 (0.17–1.71)	<0.001	
**Cancer**										
Yes	1.00	1.21 (0.96–1.52)	1.77 (1.30–2.41)	<0.001	0.009	1.00	1.11 (0.86–1.41)	1.08 (0.74–1.57)	0.698	<0.001
No	1.00	1.31 (1.12–1.53)	2.01 (1.62–2.49)	<0.001		1.00	1.16 (1.00–1.34)	1.50 (1.22–1.84)	<0.001	

*Values are weighted hazard ratio (95% confidence interval) unless stated otherwise*.

*WtHR, waist-to-height ratio; WC, waist circumference; GED, general equivalency diploma*.

*WtHR: Tertile 1, ≤ 0.54; Tertile 2, 0.55-0.62; Tertile 3, ≥0.63*.

*WC: Tertile 1, Men ≤ 0.93 cm, Women ≤ 0.88cm; Tertile 2, Men 0.94-1.05 cm, Women 0.89-1.02 cm; Tertile 3, Men ≥1.06cm, Women ≥1.03 cm*.

Two sensitivity analyses were performed to validate the findings. First, when we removed participants who had <2 years of follow-up time in the lag analysis, the results were nearly unchanged for all-cause mortality (WtHR: tertile 2, 1.28 [1.11–1.48]; tertile 3, 1.92 [1.58–2.33]; WC: tertile 2, 1.19 [1.03–1.37]; tertile 3, 1.39 [1.13–1.70]). Secondly, excluding participants with chronic conditions at baseline, the effect sizes between WC Tertile 3 or WtHR and all-cause mortality were even stronger (WtHR: tertile 2, 1.34 [1.03–1.75]; tertile 3, 2.55 [1.72–3.76]; WC: tertile 3, 1.76 [1.21–2.55]),however, the association between WC tertile 2 and all-cause mortality was no longer significant (1.11 [0.86–1.43]).

## Discussion

### Summary of Findings

In the present large prospective study of a nationally-representative cohort of U.S. adults, we showed that central obesity, as determined by the WtHR and WC, is associated with an increased risk of all-cause and CVD-related mortality, especially heart diseases-related mortality, independent of demographics, lifestyle factors, and BMI. In addition, the WtHR and WC were shown to be associated with all-cause mortality in an S-shaped non-linear and a linear dose-responsive manner, respectively, and a WtHR> 0.58 or WC > 0.98 m was shown to be a risk factor for all-cause mortality. The distribution of WtHR and WC appeared statistically significantly different between different categories of BMI (Table 1), which supported the differences between BMI and central obesity measures, therefore further corroborating the importance of measuring central obesity for the risk assessment of mortality. Our findings underscore the importance of decreasing abdominal fat accumulation to avoid central obesity, even among adults with a normal BMI, for reducing mortality risk in later life.

### Comparison With Previous Studies

Our findings are generally consistent with several previous studies (7, 13, 17). A recent dose-response meta-analysis involving 72 prospective cohort studies showed that indices of central fatness were positively and significantly associated with a higher all-cause mortality risk (17). Another meta-regression analysis involving 18 prospective studies suggested a J-shaped association between abdominal obesity, as measured by WC, and all-cause mortality (7). A meta-analysis of 82,864 participants from 9 cohort studies showed that a 1 standard deviation increase in the WHR and WC was related to a higher risk of CVD-related mortality (HR [95% CI]: 1.15 [1.05–1.25] and 1.15 [1.04–1.27], respectively) after adjusting for potential confounders (25). These findings were similar to the findings herein; however, several studies obtained different results (9, 10). One such study was a 22-years of prospective population-based cohort study from Netherlands that shown WC and the WtHR are not associated with CVD, cancer or all-cause mortality (10).Of note, the negative relationship may be due to the small sample size; only 6,366 (62.3%) persons were included in the analysis because of missing data or withdrawal (10). Additionally, the subjects were older adults (> 55 years of age) who had a higher prevalence of baseline life-threatening conditions compared with young adults or the general population; however, the information on the disease conditions at baseline were not collected and adjusted, which may have led to reverse causality for the results. Another 22-years of prospective cohort study involving 15,582 participants from China reported that central obesity was associated with lower all-cause mortality in females >60 years of age (9).The heterogeneity of results might be a reflection of different leading causes of death among the Chinese and Americans. Indeed, obesity-related CVD is the leading cause of death in the U.S. population (26), while underweight-related morbidities, such as cancer and respiratory diseases are the major causes of death in China during the study period (27). Thus, the Chinese cohort had an opposite association between central obesity and mortality in older females. Furthermore, the Chinese study did not remove participants with major diseases at baseline, and did not adjust for the history of diseases, which may have introduced confounding bias for the association.

### Interpretation of Results and Implications

There are several possible explanations for our findings. First, central obesity, reflected mainly by a large WC or WtHR, is highly associated with detrimental visceral fat and is a reflection of visceral fat accumulation (17). Excessive visceral fat is related to a variety of adverse metabolic outcomes, including insulin resistance, hyperinsulinemia, hypertension, dyslipidemia, and inflammation, which are known risk factors for CVDs and cancer (11, 28, 29). Second, the association between central obesity and mortality might reflect the characteristics of subjects with abdominal adiposity. It is possible that those individuals might be sedentary or consume more alcohol, which are confirmed risk factors for all-cause mortality (30, 31). Third, central obesity measured by a large WtHR suggests a larger WC or shorter height, or both. Previous studies have reported that taller height is inversely associated with cardiometabolic risk, such as a lower blood cholesterol concentration and systolic blood pressure, a lower plasma glucose levels, and decreased insulin resistance, which might be cardioprotective (32, 33). Thus, central obesity characterized by an increased WtHR or shorter height, resulted in high morbidity and mortality due to CVD.

Our findings may have significant clinical and public health implications. According to the 2013 AHA/ACC/TOS guideline for obesity, clinicians are recommended to use a BMI ≥ 25 kg/m^2^ as cut-off point to identify patients who need to lose weight, because the WC is measured only in overweight and obese adults (34). Thus, normal weight patients with central obesity are considered free of any particular adiposity-related risk and are not given advice or enrolled in intervention programs to lose weight. Our findings suggest that the guidelines for obesity need to be updated to recognize the potential high-risk subgroup population. In addition, public education and promotion by the Center for Disease Control and Prevention is necessary to guide people with central obesity to exercise more, change the sedentary lifestyle, and restrict diet to reduce calorie intake to decrease abdominal fat.

### Strengths and Limitations

This study had several strengths. First, the study was a prospective cohort study that used a nationally-representative sample, which facilitated generalization of the findings to U.S. adults. Second, the anthropometric data, including WC, weight, and height, were measured using precise instruments by trained staff rather than self-report, which reduced information bias. Third, a variety of demographic, lifestyle, and chronic conditional factors were available during each wave, thus we were able to control these potential confounding factors in the analyses.

Our study also had several limitations. First, anthropometric data were only measured at baseline, so we were unable to evaluate the effects of changes in central obesity during follow-up evaluations on all-cause and cause-specific mortality. Second, a total of 10,224 subjects were excluded because of pregnancy or missing data on exposure or covariates, which may have introduced bias if there were differences between those excluded and included. Third, although we adjusted for 13 potential confounders, unmeasured confounding by unmeasured variables cannot be entirely ruled out. Fourth, hip circumferences were not collected in the NHANES study, thus we cannot assess the association between the WHR and mortality. Fifth, participants who had a follow-up time of <2 years or with any chronic health conditions at baseline were excluded in the sensitivity analysis, thus reverse causation could be partially, but not completely overcome. Finally, the number of deaths cases in some subgroups such as the obese subjects in WtHR or WC tertile 1 was insufficient to generate precise estimations.

## Conclusions

This prospective cohort study of U.S. adults showed that central obesity is significantly associated with an increased risk of all-cause and CVD mortality, especially heart diseases-related mortality, even among normal adults, as weight measured by the BMI. In addition to weight control, guideline designers should provide advice and intervention programs for people to decrease abdominal fat and avoid central obesity, in an effort to reduce mortality risk in later life.

## Data Availability Statement

Publicly available datasets were analyzed in this study. This data can be found here: https://www.cdc.gov/nchs/nhanes/.

## Ethics Statement

This National Health and Nutrition Examination Survey was reviewed and approved by the Ethics Review Board at the National Center for Health Statistics (NCHS). The patients/participants and proxies provided their written informed consent to participate in this study.

## Author Contributions

PH and W-QL: conceived and design the study. JL and XY: performed the acquisition of data. PH, XY, and W-QL: conducted the statistical analysis of data. PH, JL, and XY: drafted the manuscript and all authors critically revised the manuscript for important intellectual content. All authors contributed to the article and approved the submitted version.

## Conflict of Interest

The authors declare that the research was conducted in the absence of any commercial or financial relationships that could be construed as a potential conflict of interest.

## Publisher's Note

All claims expressed in this article are solely those of the authors and do not necessarily represent those of their affiliated organizations, or those of the publisher, the editors and the reviewers. Any product that may be evaluated in this article, or claim that may be made by its manufacturer, is not guaranteed or endorsed by the publisher.

## Supplementary Material

Supplementary Figure 1 Associationsbetwen the WtHR and WC wtih all-cause and cause-specific mortality in the normal weight subgroup. Legend: Points represent the HRs, and the transverse lines represent the 95% CIs. CVD, cardiovascular disease; WtHR, waist-to-height ratio; WC, waist circumference (meter).

Supplementary Figure 2 Associations betwen the WtHR and WC wtih all-cause and cause-specific mortality in the overweight subgroup. Legend: Points represent the HRs, and the transverse lines represent the 95% CIs. CVD, cardiovascular disease; WtHR, waist-to-height ratio; WC, waist circumference (meter).

## References

[1] Wang Y Beydoun MA Min J Xue H Kaminsky LA Cheskin LJ. Has the prevalence of overweight, obesity and central obesity levelled off in the United States? Trends, patterns, disparities, and future projections for the obesity epidemic. Int J Epidemiol. (2020) 49:810–23. 10.1093/ije/dyz273 32016289 PMC7394965

[2] Ford ES Maynard LM Li C. Trends in mean waist circumference and abdominal obesity among US adults, 1999-2012. JAMA. (2014) 312:1151–3. 10.1001/jama.2014.8362 25226482 PMC4608432

[3] Chen GC Arthur R Iyengar NM Kamensky V Xue X Wassertheil-Smoller S. Association between regional body fat and cardiovascular disease risk among postmenopausal women with normal body mass index. Eur Heart J. (2019) 40:2849–55. 10.1093/eurheartj/ehz391 31256194 PMC6933870

[4] Yu D Zheng W Johansson M Lan Q Park Y White E. Overall and central obesity and risk of lung cancer: a pooled analysis. J Natl Cancer Inst. (2018) 110:831–42. 10.1093/jnci/djx286 29518203 PMC6093439

[5] Pang Y Kartsonaki C Guo Y Chen Y Yang L Bian Z. Central adiposity in relation to risk of liver cancer in Chinese adults: A prospective study of 05 million people. Int J Cancer. (2019) 145:1245–53. 10.1002/ijc.32148 30665257 PMC6767784

[6] Zhang C Rexrode KM van Dam RM Li TY Hu FB. Abdominal obesity and the risk of all-cause, cardiovascular, and cancer mortality: sixteen years of follow-up in US women. Circulation. (2008) 117:1658–67. 10.1161/CIRCULATIONAHA.107.739714 18362231

[7] Carmienke S Freitag MH Pischon T Schlattmann P Fankhaenel T Goebel H. General and abdominal obesity parameters and their combination in relation to mortality: a systematic review and meta-regression analysis. Eur J Clin Nutr. (2013) 67:573–85. 10.1038/ejcn.2013.61 23511854

[8] Pischon T Boeing H Hoffmann K Bergmann M Schulze MB Overvad K. General and abdominal adiposity and risk of death in Europe. N Engl J Med. (2008) 359:2105–20. 10.1056/NEJMoa0801891 19005195

[9] Chen Y Yang Y Jiang H Liang X Wang Y Lu W. Associations of BMI and waist circumference with all-cause mortality: a 22-year cohort study. Obesity. (2019) 27:662–9. 10.1002/oby.22423 30861324

[10] Dhana K Kavousi M Ikram MA Tiemeier HW Hofman A Franco OH. Body shape index in comparison with other anthropometric measures in prediction of total and cause-specific mortality. J Epidemiol Commun Health. (2016) 70:90–6. 10.1136/jech-2014-205257 26160362

[11] Sun Y Liu B Snetselaar LG Wallace RB Caan BJ Rohan TE. Association of normal-weight central obesity with all-cause and cause-specific mortality among postmenopausal women. JAMA Netw Open. (2019) 2:e197337. 10.1001/jamanetworkopen.2019.7337 31339542 PMC6659146

[12] Liu L Gao B Wang J Yang C Wu S Wu Y. Joint association of body mass index and central obesity with cardiovascular events and all-cause mortality in prediabetic population: A prospective cohort study. Obes Res Clin Pract. (2019) 13:453–61. 10.1016/j.orcp.2019.08.004 31558371

[13] Sharma S Batsis JA Coutinho T Somers VK Hodge DO Carter RE. Normal-weight central obesity and mortality risk in older adults with coronary artery disease. Mayo Clin Proc. (2016) 91:343–51. 10.1016/j.mayocp.2015.12.007 26860580

[14] Gnatiuc L Alegre-Díaz J Wade R Ramirez-Reyes R Tapia-Conyer R Garcilazo-Ávila A. General and abdominal adiposity and mortality in mexico city: a prospective study of 150 000 adults. Ann Intern Med. (2019) 171:397–405. 10.7326/M18-3502 31404923 PMC6949137

[15] Roswall N Li Y Sandin S Ström P Adami HO Weiderpass E. Changes in body mass index and waist circumference and concurrent mortality among Swedish women. Obesity. (2017) 25:215–22. 10.1002/oby.21675 27768253

[16] Connor GS Tremblay M Moher D Gorber B. A comparison of direct vs. self-report measures for assessing height, weight and body mass index: a systematic review. Obes Rev. (2007) 8:307–26. 10.1111/j.1467-789X.2007.00347.x 17578381

[17] Jayedi A Soltani S Zargar MS Khan TA Shab-Bidar S. Central fatness and risk of all cause mortality: systematic review and dose-response meta-analysis of 72 prospective cohort studies. BMJ. (2020) 370:m3324. 10.1136/bmj.m3324 32967840 PMC7509947

[18] Centers for Disease Control and Prevention. Available online at: https://www.cdc.gov/nchs/nhanes/ (accessed July 16, 2021).

[19] National Center for Health Statistics Centers for Disease Control and Prevention. National Health and Nutrition Examination Survey (NHANES) Anthropometry Procedures Manual. (2009). Available online at: https://wwwn.cdc.gov/nchs/data/nhanes/2009-2010/manuals/bodymeasures_09.pdf (accessed July 16, 2021).

[20] National Center for Health Statistics Centers for Disease Control and Prevention. 2015 Public-Use Linked Mortality Files. Available online at: https://www.cdc.gov/nchs/data-linkage/mortality-public.htm (accessed July 16, 2021).

[21] National Center for Health Statistics Centers for Disease Control Prevention. NHANES I Epidemiologic Follow-up Survey (NHEFS) Calibration Sample for NDI Matching Methodology. (2009). Available online at: https://www.cdc.gov/nchs/data/datalinkage/mort_calibration_study.pdf (Accessed July 16, 2021).

[22] Ganna A Ingelsson E. 5 year mortality predictors in 498,103 UK Biobank participants: a prospective population-based study. Lancet. (2015) 386:533–40. 10.1016/S0140-6736(15)60175-1 26049253

[23] Liangpunsakul S. Relationship between alcohol intake and dietary pattern: findings from NHANES III. World J Gastroenterol. (2010) 16:4055–60. 10.3748/wjg.v16.i32.4055 20731019 PMC2928459

[24] National Center for Health Statistics Centers for Disease Control Prevention. NHANES Survey Methods and Analytic Guidelines. Available online at: https://wwwn.cdc.gov/nchs/nhanes/analyticguidelines.aspx#estimation-and-weighting-procedures. (accessed July 16, 2021).

[25] Czernichow S Kengne AP Stamatakis E Hamer M. Batty GD. Body mass index, waist circumference and waist-hip ratio: which is the better discriminator of cardiovascular disease mortality risk?: evidence from an individual-participant meta-analysis of 82 864 participants from nine cohort studies. Obes Rev. (2011) 12:680–7. 10.1111/j.1467-789X.2011.00879.x 21521449 PMC4170776

[26] Roth GA Johnson CO Abate KH Abd-Allah F Ahmed M Alam K. The burden of cardiovascular diseases among US States, 1990-2016. Jama Cardiol. (2018) 3:375–89. 10.1001/jamacardio.2018.0385 29641820 PMC6145754

[27] Lian Z Xie Y Lu Y Huang D Shi H. Trends in the major causes of death in China, 1982-2010. Chin Med J. (2014) 127:777–81. 10.3760/cma.j.issn.0366-6999.20131054 24534240

[28] Patel TP Rawal K Bagchi AK Akolkar G Bernardes N Dias DDS. Insulin resistance: an additional risk factor in the pathogenesis of cardiovascular disease in type 2 diabetes. Heart Fail Rev. (2016) 21:11–23. 10.1007/s10741-015-9515-6 26542377

[29] Deng T Lyon CJ Bergin S Caligiuri MA Hsueh WA. Obesity, Inflammation, and Cancer. Annu Rev Pathol. (2016) 11:421–49. 10.1146/annurev-pathol-012615-044359 27193454

[30] Zhao M Veeranki SP Magnussen CG Xi B. Recommended physical activity and all cause and cause specific mortality in US adults: prospective cohort study. BMJ. (2020) 370:m2031. 10.1136/bmj.m2031 32611588 PMC7328465

[31] Xi B Veeranki SP Zhao M Ma C Yan Y. Mi J. Relationship of Alcohol Consumption to All-Cause, Cardiovascular, and Cancer-Related Mortality in US Adults J Am Coll Cardiol. (2017) 70:913–22. 10.1016/j.jacc.2017.06.054 28818200

[32] Schulze MB Stefan N. Genetic Predisposition to Abdominal Adiposity and Cardiometabolic Risk. JAMA. (2017) 317:2334. 10.1001/jama.2017.5041 28609526

[33] Stefan N Häring HU Hu FB Schulze MB. Divergent associations of height with cardiometabolic disease and cancer: epidemiology, pathophysiology, and global implications. Lancet Diabetes Endocrinol. (2016) 4:457–67. 10.1016/S2213-8587(15)00474-X 26827112

[34] Jensen MD Ryan DH Apovian CM Ard JD Comuzzie AG Donato KA. 2013 AHA/ACC/TOS guideline for the management of overweight and obesity in adults: a report of the American College of Cardiology/American Heart Association Task Force on Practice Guidelines and The Obesity Society. Circulation. (2014) 129:S102–38. 10.1161/01.cir.0000437739.71477.ee 24222017 PMC5819889

