# Supplementary figures and images for: Association of Central Obesity With All Cause and Cause-Specific Mortality in US Adults: A Prospective Cohort Study
Source: Front Cardiovasc Med. 2022 Jan 28;9:816144. doi: 10.3389/fcvm.2022.816144 (PMC8832149; doi:10.3389/fcvm.2022.816144)

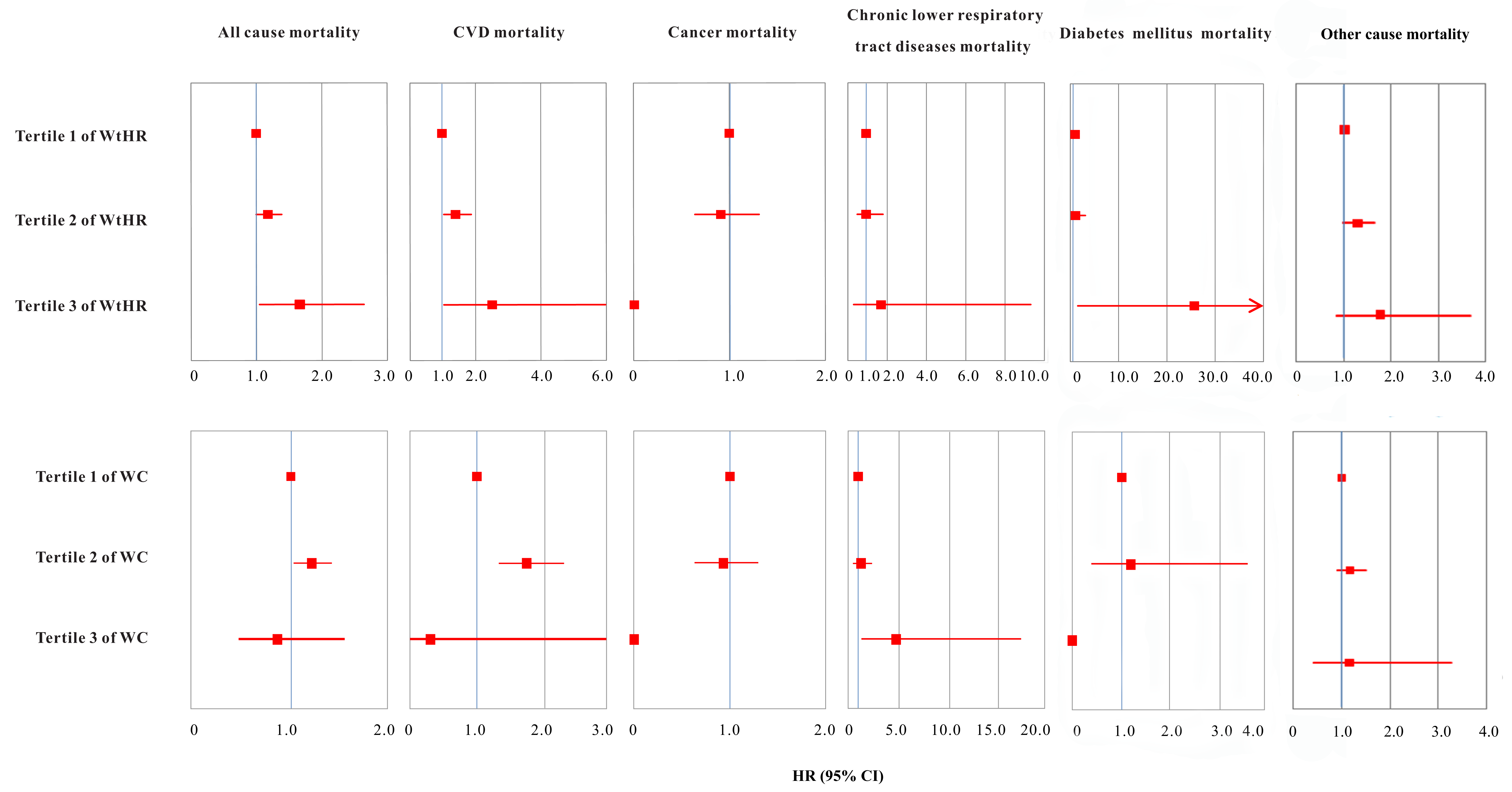

Supplement: Supplementary Figure 1 — Associationsbetwen the WtHR and WC wtih all-cause and cause-specific mortality in the normal weight subgroup. Legend: Points represent the HRs, and the transverse lines represent the 95% CIs. CVD, cardiovascular disease; WtHR, waist-to-height ratio; WC, waist circumference (meter). [file Image_1.TIF]

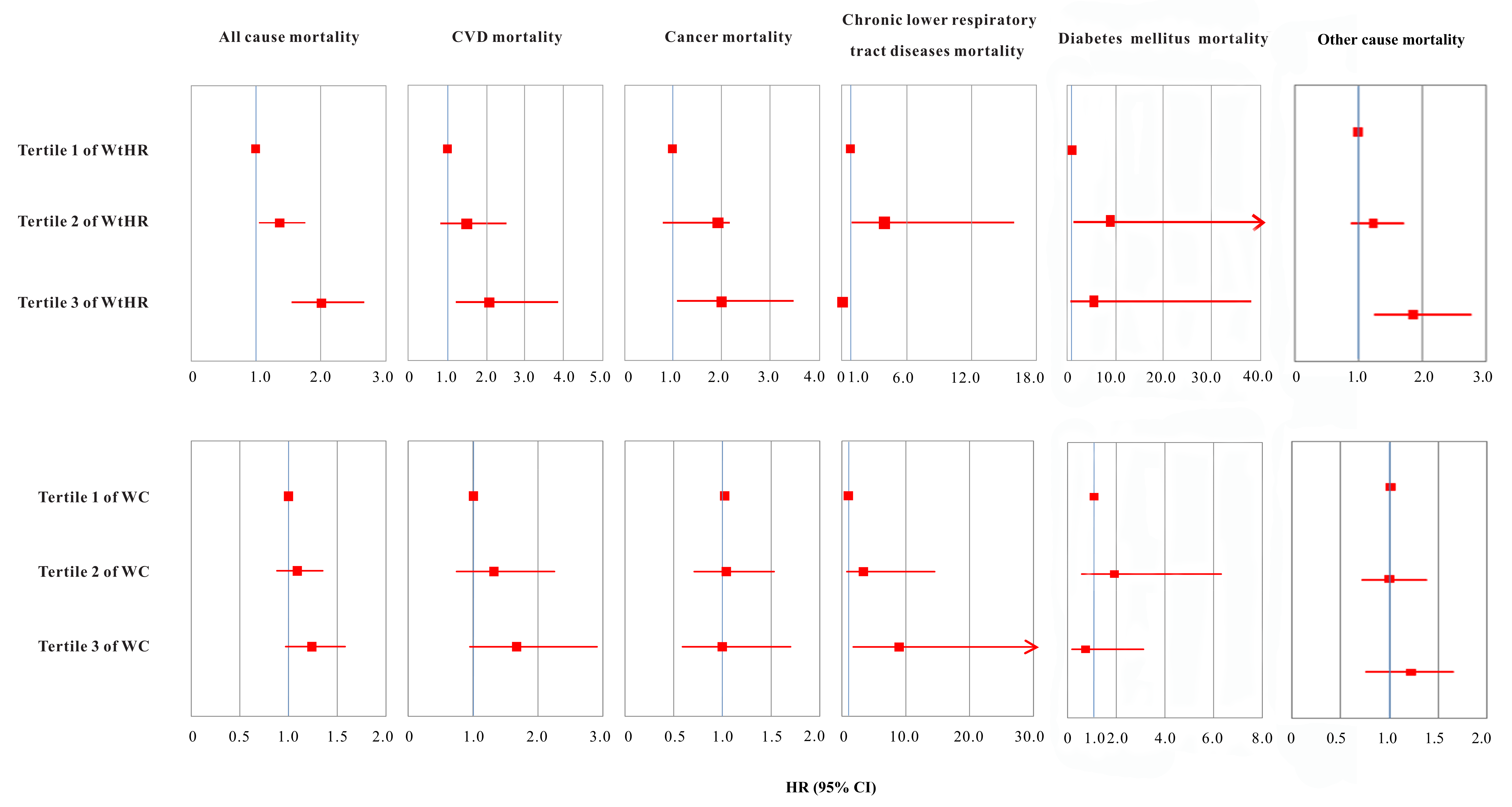

Supplement: Supplementary Figure 2 — Associations betwen the WtHR and WC wtih all-cause and cause-specific mortality in the overweight subgroup. Legend: Points represent the HRs, and the transverse lines represent the 95% CIs. CVD, cardiovascular disease; WtHR, waist-to-height ratio; WC, waist circumference (meter). [file Image_2.TIF]
